# Reply to: Pitfalls in the genetic testing of the *OPN1LW*-*OPN1MW* gene cluster in human subjects

**DOI:** 10.1038/s41525-024-00409-9

**Published:** 2024-05-04

**Authors:** Lonneke Haer-Wigman, Amber den Ouden, Ronny Derks, Maria M. van Genderen, Dorien Lugtenberg, Joke Verheij, Raymon Vijzelaar, Helger G. Yntema, Lisenka E. L. M. Vissers, Kornelia Neveling

**Affiliations:** 1 Department of Human Genetics, Radboud University Medical Center, Nijmegen, The Netherlands; 2 Research Institute for Medical Innovation, Radboud University Medical Center, Nijmegen, The Netherlands; 3 Bartiméus Diagnostic Center for complex visual disorders, Zeist, the Netherlands; 4 Department of Ophthalmology, University Medical Centre Utrecht, Utrecht, the Netherlands; 5 Department of Genetics, University Medical Center Groningen, University of Groningen, Groningen, the Netherlands; 6 MRC Holland b v, Amsterdam, the Netherlands

**Keywords:** Vision disorders, Molecular medicine

**replying to** Wissinger, B. *npj* *Genomic Medicine* 10.1038/s41525-024-00406-y (2024)

We recently published a genetic assay for the *OPN1LW*/*OPN1MW* gene cluster, comprising four amplicon-based long-read sequencing reactions and one MLPA^1^. Wissinger et al. brought to our attention that, for one of the four amplicons, the primer overlaps with a polymorphism, designated SDIns^2^. Consequently, the amplicon would be non-specific, from which they claim that our approach will render misleading results. We performed several analyses to disprove this claim. Firstly, it could only lead to wrong results if this amplicon requires assessment. In practice, retrospectively evaluated from 155 male probands, this amplicon requires analysis in only 8% of probands. Moreover, errors in the genetic composition would require a certain distribution of SDIns across the *OPN1LW*/*OPN1MW* cluster, which, based on the analysis of 200 alleles using optical genome mapping, does not seem to occur in the population. Indeed, long read genome sequencing, in combination with de novo assembly for two cases from the original publication, confirmed our original findings. Lastly, it is not always necessary to know the complete composition of the cluster to conclude whether or not the clinical diagnosis is genetically confirmed. In all 8% probands in whom this amplicon would be analyzed an incorrect determination of the composition of the cluster would have resulted in the same conclusion of the genetic assay. Taken these results together, the genetic assay for the *OPN1LW*/*OPN1MW* gene cluster as described in our previous paper^1^ allows for a clinically relevant determination of the composition of the *OPN1LW*/*OPN1MW* gene cluster.

With interest, we took notice of the manuscript by Wissinger and colleagues^2^. Indeed, as highlighted by the authors, the reverse primer to amplify the last opsin gene copy is located within the segmental duplicated region of *OPN1MW*. This segmental duplication region is, however, interrupted by non-repeat sequence (Fig. 1): a 697 base pair insertion polymorphism (denoted as SDIns by Wissinger et al.). Although this SDIns is exclusively reported to be downstream of the second *OPN1MW* gene copy in all three available reference genomes GRCh37/hg19, GRCh38 and T2T-CHM13, Ueyama et al. already published in 2004 its occurrence and distribution across the opsin gene cluster in detail and highlighted the benefits of using the SDIns for characterization of the *OPN1LW*/*OPN1MW* cluster^3^. Wissinger et al. confirms that this SDIns is not exclusively located downstream of the last opsin gene copy^2^, but challenges its use to characterize the *OPN1LW*/*OPN1MW* cluster because of non-specific amplification, and thus also challenges our approach.

**Fig. 1:**
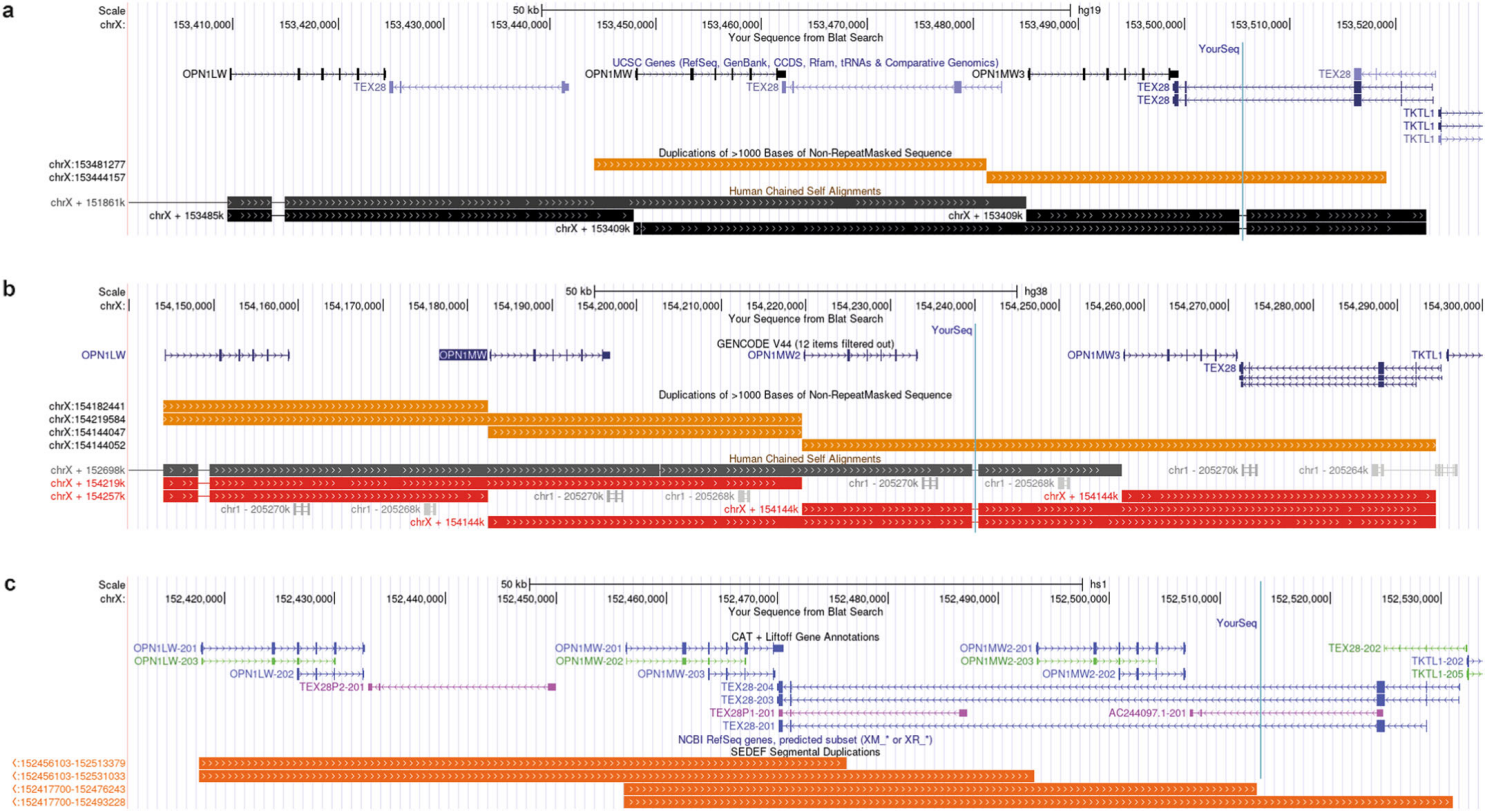
Print screens of UCSC genome track of the *OPN1LW/OPN1MW* gene cluster in different genome assemblies. Three different genome assemblies are shown, GRCh37/hg19 in (**a**), GRCh38 in (**b**), and T2T in (**c**). In all three panels, the location of the reverse primers sequence (099-821, TCTCATTCATAAATTGCTGGTA) of the amplicon for the last opsin gene in the cluster is depicted by the light blue bar. Although the primer sequence is located within the segmental duplicated region in the GRCh37/hg19, GRCh38, and T2T assemblies, the primer sequence is uniquely aligned in all three genome builds. The human chained self-alignment track (only available for GRch37/hg19 and GRCh38 assemblies) visualizes the fact that the segmental duplication is interrupted and the primer is located in the non-repeat region. In GRCh37 and T2T, the *OPN1LW/OPN1MW* gene cluster consists of three opsin gene copies and primer 099-821 is located behind the last opsin gene copy of the cluster (**a**, **c**), while in GRCh38, the *OPN1LW/OPN1MW* gene cluster consists of four opsin gene copies and primer 099-821 is located behind the second-last opsin gene copy of the cluster (**b**).

In our approach^1^, the amplicon for the last opsin gene copy only requires sequencing in patients with three opsin gene copies in whom the last two opsin gene copies are not identical, as in patients with three or more opsin gene copies in which the second and consecutive gene copies are identical, the exact order is redundant. Also, as already stated our approach cannot determine the composition of the complete cluster when the cluster contains four or more opsin gene copies and the second and consecutive gene copies are not identical^1^. We aimed to assess the impact of Wissinger et al.’s notions by determining the number of patients in his cohort fulfilling the above criteria, but cannot do so as essential information on the complete make-up of the *OPN1LW*/*OPN1MW* gene cluster of the samples in their cohort and detailed methods are lacking^2^. We therefore revisited the genetic test results of a cohort of 155 male probands for whom genetic testing of the *OPN1LW*/*OPN1MW* gene cluster was performed, including 33 of our original report^1^. Of these 155 patients, 70 (45%) had three or more opsin gene copies in their cluster, but only 13 (8%) had three opsin copies where the last two copies differed, putting them at risk for errors in the genetic outcome of our approach (Supplementary Table 1, Supplementary Fig. 1).

To comprehensively assess this risk, it is, however—*in addition to the absolute copy number of SDIns*—also essential to understand the distribution of SDIns over the different opsin gene copies. Whereas Wissinger et al. determined the copy number of the SDIns using qPCR, positional information cannot be determined from their approach^2^, as this would require single long molecules spanning the distance of the entire *OPN1LW*/*OPN1MW* cluster. We, therefore, used optical genome mapping^4^ to determine the location of SDIns in 200 alleles from random individuals (color vision status unknown) with three opsin gene copies (Supplemental Fig. 3). This analysis showed that in the vast majority of alleles (153 of 200; 76.5%), SDIns was located solely after the last opsin gene copy (Fig. 2a); in 12 alleles (6%), SDIns was present after the first and last opsin gene copy (Fig. 2b). For both situations, collectively representing 82.5% of individuals with three copies of the opsin cluster, a correct diagnosis would be obtained using our originally reported approach. In 35 alleles (17.5%) the amplicon would render an inconclusive genetic result due to overlapping sequences or no PCR products (Fig. 2c–f). Most importantly, the two possible options that would lead to an incorrect genetic result, e.g. the presence of the SDIns after the second opsin gene copy and the absence of the SDIns after the third opsin gene copy, were absent from the 200 investigated alleles (Fig. 2g, h). Therefore, extrapolating these results from the population suggests that the likelihood of an incorrect determination of the composition of the cluster is negligible. However, as the majority of alleles analyzed is probably from individuals of a Caucasian background, there is a possibility that the distribution of the SDIns differs in populations with other ethnicities. Of note, as an additional verification, we performed long-read genome sequencing for two of the clinical cases from the original study^1^, which for both confirmed the genetic composition of the *OPN1LW*/*OPN1MW* gene cluster as reported (Supplemental Fig. 2).

**Fig. 2:**
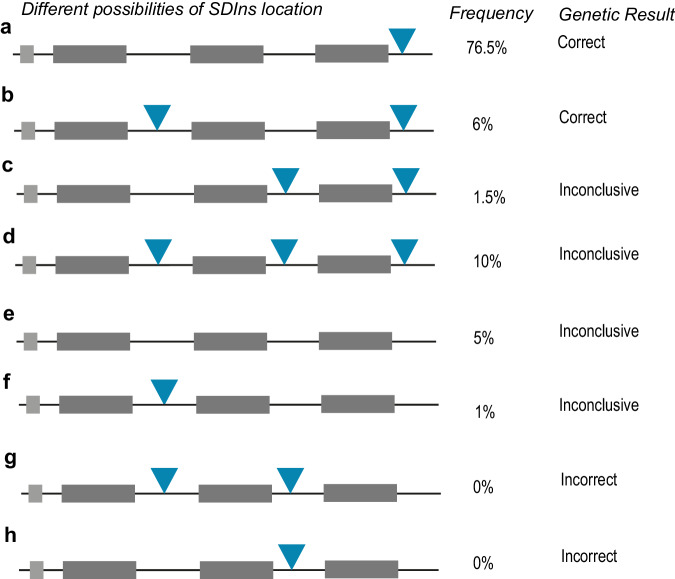
Schematic representation of all possible locations of the SDIns (blue triangle) in a three opsin gene copy (dark grey bars) cluster. The figure shows the frequency of the specific composition determined in 200 alleles of individuals (both male and female) whose color vision status was unknown, plus whether the specific composition would lead to a correct inconclusive or incorrect result. **a** In 76.5% (135 alleles) the SDIns are solely located behind the last opsin gen copy and the genetic assay would correctly determine the composition of the cluster. **b** In 6% (12 alleles) the polymorphism can be found downstream of the first and of the last gene. Also, for this situation, the genetic assay would correctly determine the composition of the genetic cluster, as with long-read sequencing one can easily differentiate between the first and last gene copy using the result of the amplicon specific for the first gene copy. In 17.5% (35 alleles) the genetic assays would not be able to determine the exact composition of the cluster as the amplicon for the last opsin gene copy would sequence both the second-to-last and last opsin gene copy **c**, **d** or neither the second-to-last and last opsin gene copy would be amplified. **e**, **f** These last four options, would not lead to an incorrect genetic diagnosis as the sequencing results clearly show an inconclusive result: either no or two alleles are sequenced. **g**, **h** The two possibilities where an incorrect genetic result would be determined with the genetic assay. This situation could, however, not be detected in 200 alleles with three opsin gene copies.

Despite the unlikely scenario of an incorrect determination of *OPN1LW*/*OPN1MW* gene cluster, we assessed whether or not this would impact the clinical diagnosis. In all 13 probands with three opsin gene copies of which the last two were different, sequencing of the amplicon for the first opsin gene copy, and the amplicon for the second and if present consecutive opsin gene copies were already enough for a conclusive test result, irrespective of it being positive, inconclusive or negative. Thus, the result for the amplicon for the last gene copy would refine the composition, but would not change the conclusion.

The discussion on strategies to evaluate the *OPN1LW*/*OPN1MW* gene cluster highlights the complexity of the locus. Whereas we agree with Wissinger et al. that, ideally, (ultra-)long read (genome) sequencing with de novo assembly would help to resolve these challenges, such strategy is not yet available in routine diagnostic settings, due to the relative high costs (long read circular consensus sequencing developed by Pacific Biosciences), or relative high per base error rate (long read nanopore sequencing developed by Oxford Nanopore)^5^. Having shown that SDIns has no impact our on diagnostic outcomes, and that analysis of the third copy of *OPN1LW*/*OPN1MW* is obsolete for clinical interpretation, we therefore remain of the opinion that at the moment, the genetic test described by Haer-Wigman et al.^1^ is the most complete diagnostic test for the *OPN1LW*/*OPN1MW* gene cluster.

## Reporting summary

Further information on research design is available in the Nature Research Reporting Summary linked to this article.

### Supplementary information

REPORTING SUMMARY

Supplementary information

## Data Availability

The individual-level sequencing and optical genome mapping data are available behind the Radboudumc firewall. In the absence of explicit data-sharing consent at the individual patient level, FASTQ, BAM, and VCFs cannot be disclosed. These data are, however, available for review at or via a secured connection with the Department of Human Genetics of the Radboudumc. Data is available upon reasonable request and after a data usage agreement through the corresponding author L.H-W.
