# Supplementary material for: Reply to: Pitfalls in the genetic testing of the OPN1LW-OPN1MW gene cluster in human subjects
Source: NPJ Genom Med. 2024 May 4;9:29. doi: 10.1038/s41525-024-00409-9 (PMC11069539; doi:10.1038/s41525-024-00409-9)
Supplement: Supplementary file 1 — REPORTING SUMMARY [file 41525_2024_409_MOESM1_ESM.pdf]

nature portfolio

Corresponding author(s): Dr Lonneke Haer-Wigman

Last updated by author(s): Feb 2, 2024

Reporting Summary

Nature Portfolio wishes to improve the reproducibility of the work that we publish. This form provides structure for consistency and transparency in reporting. For further information on Nature Portfolio policies, see our Editorial Policies and the Editorial Policy Checklist.

Statistics

For all statistical analyses, confirm that the following items are present in the figure legend, table legend, main text, or Methods section.

n/a	Confirmed	
☐	☒	The exact sample size (*n*) for each experimental group/condition, given as a discrete number and unit of measurement
☐	☒	A statement on whether measurements were taken from distinct samples or whether the same sample was measured repeatedly
☒	☐	The statistical test(s) used AND whether they are one- or two-sided<br>*Only common tests should be described solely by name; describe more complex techniques in the Methods section.*
☒	☐	A description of all covariates tested
☒	☐	A description of any assumptions or corrections, such as tests of normality and adjustment for multiple comparisons
☒	☐	A full description of the statistical parameters including central tendency (e.g. means) or other basic estimates (e.g. regression coefficient) AND variation (e.g. standard deviation) or associated estimates of uncertainty (e.g. confidence intervals)
☒	☐	For null hypothesis testing, the test statistic (e.g. *F*, *t*, *r*) with confidence intervals, effect sizes, degrees of freedom and *P* value noted<br>*Give P values as exact values whenever suitable.*
☒	☐	For Bayesian analysis, information on the choice of priors and Markov chain Monte Carlo settings
☒	☐	For hierarchical and complex designs, identification of the appropriate level for tests and full reporting of outcomes
☒	☐	Estimates of effect sizes (e.g. Cohen's *d*, Pearson's *r*), indicating how they were calculated

Our web collection on statistics for biologists contains articles on many of the points above.

Software and code

Policy information about availability of computer code

Data collection	MLPA samples were analyzed on a fragment analyse (Model 31310, Applied Biosystems). Long-read amplicon sequencing was performed using the Run Design option in SMRTLNK, and was performed on a Sequel I or Sequel II2 system (Pacific Biosciences) with ICS 6.0. Optical genome mapping was performed on the Saphyr System (Bionano Genomics) using ICS version 5.2. Long read genome sequencing was performed using PacBio HiFi sequencing (Pacific Biosciences).
Data analysis	Genemarker (V2.6.7, Softgentics) was used for data analysis of MLPA results. Long-read sequencing data was analuzed using CC mapping in SMRTLink. Generated BAM files were uploaded in SeqNect (JSI Medical systems) and variant calling was performed using default settings on OPN1LW (NM_020061.6) and OPN1MW (NM_000513.2) or via an in-house developed pipeline and analysis software. The annotated de novo assembly pipeline was executed with Bionano Solve software 3.6.1 was performed on optical genome mapping results. Long-read genome analysis was performed in IGV.

For manuscripts utilizing custom algorithms or software that are central to the research but not yet described in published literature, software must be made available to editors and reviewers. We strongly encourage code deposition in a community repository (e.g. GitHub). See the Nature Portfolio guidelines for submitting code & software for further information.

Data

Policy information about availability of data

All manuscripts must include a data availability statement. This statement should provide the following information, where applicable:

- Accession codes, unique identifiers, or web links for publicly available datasets
- A description of any restrictions on data availability
- For clinical datasets or third party data, please ensure that the statement adheres to our policy

The individual-level sequencing and optical genome mapping data are available behind the Radboudumc firewall. In absence of explicit data sharing consent at individual patient level, FASTQ, BAM and VCFs cannot be disclosed. These data are, however, available for review at or via a secured connection with the department of Human Genetics of the Radboudumc. Data is available upon reasonable request and after a data usage agreement through corresponding author L.H-W.

Research involving human participants, their data, or biological material

Policy information about studies with human participants or human data. See also policy information about sex, gender (identity/presentation), and sexual orientation and race, ethnicity and racism.

Reporting on sex and gender	As the OPN1LW-OPN1MW gene cluster is located on the X-chromosome it was relevant to use the term male.
Reporting on race, ethnicity, or other socially relevant groupings	Not applicable
Population characteristics	Male patient with a clinical question regarding the OPN1LW/OPN1MW gene cluser were included in this study.
Recruitment	Genetic analysis of the OPN1LW/OPN1MW gene cluster was requested for 155 probands between 2015 and September 2023 at the Department of Human Genetics of the Radboud University Medical Center. 229 random anonymized samples on whom optical genome mapping was performed were selected.
Ethics oversight	Genetic analysis of the OPN1LW/OPN1MW gene cluster was requested for 155 probands between 2015 and September 2023 at the Department of Human Genetics of the Radboud University Medical Center. 229 random anonymized samples on whom optical genome mapping was performed were selected.

Note that full information on the approval of the study protocol must also be provided in the manuscript.

Field-specific reporting

Please select the one below that is the best fit for your research. If you are not sure, read the appropriate sections before making your selection.

☒ Life sciences ☐ Behavioural & social sciences ☐ Ecological, evolutionary & environmental sciences

For a reference copy of the document with all sections, see nature.com/documents/nr-reporting-summary-flat.pdf

Life sciences study design

All studies must disclose on these points even when the disclosure is negative.

Sample size	155 probands and 229 control samples
Data exclusions	No data was excluded
Replication	No replication was performed
Randomization	No randomization was performed
Blinding	No blinding was done

Reporting for specific materials, systems and methods

We require information from authors about some types of materials, experimental systems and methods used in many studies. Here, indicate whether each material, system or method listed is relevant to your study. If you are not sure if a list item applies to your research, read the appropriate section before selecting a response.

Materials & experimental systems

n/a	Involved in the study
☒	☐ Antibodies
☒	☐ Eukaryotic cell lines
☒	☐ Palaeontology and archaeology
☒	☐ Animals and other organisms
☒	☐ Clinical data
☒	☐ Dual use research of concern
☒	☐ Plants

Methods

n/a	Involved in the study
☒	☐ ChIP-seq
☒	☐ Flow cytometry
☒	☐ MRI-based neuroimaging

Plants

Seed stocks	*Report on the source of all seed stocks or other plant material used. If applicable, state the seed stock centre and catalogue number. If plant specimens were collected from the field, describe the collection location, date and sampling procedures.*
Novel plant genotypes	*Describe the methods by which all novel plant genotypes were produced. This includes those generated by transgenic approaches, gene editing, chemical/radiation-based mutagenesis and hybridization. For transgenic lines, describe the transformation method, the number of independent lines analyzed and the generation upon which experiments were performed. For gene-edited lines, describe the editor used, the endogenous sequence targeted for editing, the targeting guide RNA sequence (if applicable) and how the editor was applied.*
Authentication	*Describe any authentication procedures for each seed stock used or novel genotype generated. Describe any experiments used to assess the effect of a mutation and, where applicable, how potential secondary effects (e.g. second site T-DNA insertions, mosiacism, off-target gene editing) were examined.*
