# Supplementary material for: Reply to: Pitfalls in the genetic testing of the OPN1LW-OPN1MW gene cluster in human subjects
Source: NPJ Genom Med. 2024 May 4;9:29. doi: 10.1038/s41525-024-00409-9 (PMC11069539; doi:10.1038/s41525-024-00409-9)
Supplement: Supplementary file 2 — Supplementary information [file 41525_2024_409_MOESM2_ESM.pdf]

SUPPLEMENTARY INFORMATION

This supplementary information includes

- Supplementary methods
- 2 Supplementary Tables
    - Supplementary Table 1: 155 male samples analyzed for the *OPN1LW/MW* gene cluster
    - Supplementary Table 2: Quality values of long-read genome sequencing
- 3 Supplementary Figures
    - Supplementary Figure 1: Flow diagram of results of the genetic analysis of the *OPN1LW/OPN1MW* gene cluster in 155 XY probands.
    - Supplementary Figure 2: Print screens of IGV of the *OPN1LW/OPN1MW* gene cluster of *de novo* assembly of long-read genome sequencing data of USN15524 and USN15600
    - Supplementary Figure 3: OGM data representing the *OPN1LW/MW* gene cluster containing one *OPN1LW* gene copy and two *OPN1MW* gene copies, as given in reference GRCh37/hg19

SUPPLEMENTARY METHODS

Genetic data from clinical archive

Genetic analysis of the *OPN1LW/OPN1MW* gene cluster was requested for 155 probands between 2015 and September 2023 at the Department of Human Genetics of the Radboud University Medical Center. In all probands genetic analysis of the *OPN1LW/OPN1MW* gene cluster was performed using MLPA and long-read sequencing of at least the amplicon for first opsin gene copy and the amplicon for the second and if present consecutive opsin gene copies as described by Haer-Wigman *et al*.(1) Of these 155 probands 33 are also described in Haer-Wigman *et al*(1) For probands included in both studies, sample numbering (USN number) has been kept identical.

Long read genome sequencing

Long-read genome sequencing was performed using PacBio HiFi sequencing (Pacific Biosciences, Menlo Park, CA, USA), using peripheral DNA isolated according to standard procedure.(2) In brief, 7-8 µg DNA was sheared on a Megaruptor 3 to a target size of 15-18 kb. Library preparation was performed using the SMRTbell Prep Kit 3.0 according to the manufacturer's instructions (Pacific Biosciences). Size selection was performed using a BluePippin system (target fragments >10 kb). Sequence primer and polymerase were bound to the complex using the Revio Polymerase Kit. Sequencing was performed on a Revio system (Pacific Biosciences, Menlo Park, CA, USA), according to the manufacturer's instructions. A *de novo* assembly was performed and data was aligned to the GRCh38 gnome build. Data analysis was performed in IGV.

Optical genome mapping

Optical genome mapping was performed as described previously.(1) The opsin gene copy number was determined in 229 random anonymized samples (100 male, 129 females; 358 alleles) in whom color vision status was unknown. In 200 of 358 alleles, three opsin gene copies were observed and thus evaluated for the distribution of SDIns using GRCh37/hg19. As these samples where anonymized the ethnic distribution of this cohort could not be determined, but we assume the majority of alleles are from individuals with a Caucasian background.

SUPPLEMENTARY TABLES

Supplementary Table 1. 155 male samples analyzed for the OPN1LW/MW gene cluster

USN	Present in Haer-Wigman et al (1)	Reason for referral	Copy number LCR	Total number of opsin gene copies	Coding sequence second, third, etc opsin gene copy	Determination of the composition of the cluster	Diagnostic conclusion of genetic assay of *OPN1LW/OPN1MW* gene cluster
**00165**	No	Bornholm eye disease	1	2	Identical	Complete	No genetic confirmation of clinical diagnosis
**00236**	No	Bornholm eye disease	1	4	Different	Incomplete	No genetic confirmation of clinical diagnosis
**00280**	No	Blue cone monochromacy	1	2	Identical	Complete	No genetic confirmation of clinical diagnosis
**00325**	Yes	Bornholm eye disease	1	2	Identical	Complete	Genetic confirmation of clinical diagnosis
**00546**	No	Bornholm eye disease	1	3	Identical	Complete	No genetic confirmation of clinical diagnosis
**00565**	No	Bornholm eye disease	1	2	Identical	Complete	Genetic confirmation of clinical diagnosis
**15600**	Yes	Blue cone monochromacy	1	3	Different	Complete	Genetic confirmation of clinical diagnosis
**00784**	No	Bornholm eye disease	1	3	Identical	Complete	No genetic confirmation of clinical diagnosis
**00839**	No	Bornholm eye disease	1	2	Identical	Complete	Genetic confirmation of clinical diagnosis
**00998**	No	Blue cone monochromacy	1	1	Identical	Complete	Genetic confirmation of clinical diagnosis
**01051**	No	Blue cone monochromacy	1	5	Identical	Complete	No genetic confirmation of clinical diagnosis
**01185**	No	Bornholm eye disease	1	2	Identical	Complete	Genetic confirmation of clinical diagnosis
**01301**	No	Bornholm eye disease	1	2	Identical	Complete	Genetic confirmation of clinical diagnosis
**01345**	Yes	Bornholm eye disease	1	3	Identical	Complete	No genetic confirmation of clinical diagnosis
**01552**	No	Bornholm eye disease	1	1	Identical	Complete	Genetic confirmation of clinical diagnosis
**20458**	Yes	Bornholm eye disease	1	3	Different	Incomplete	Genetic confirmation of clinical diagnosis
**02090**	No	Cone dystrophy	1	3	Identical	Complete	Genetic confirmation of clinical diagnosis
**02170**	Yes	Bornholm eye disease	1	1	Identical	Complete	Genetic confirmation of clinical diagnosis
**13114**	No	Blue cone monochromacy	1	3	Different	Complete	Genetic confirmation of clinical diagnosis
**02376**	No	Bornholm eye disease	1	2	Identical	Complete	Genetic confirmation of clinical diagnosis
**02393**	Yes	Bornholm eye disease	1	3	Identical	Complete	Genetic confirmation of clinical diagnosis
**02577**	No	Bornholm eye disease	1	2	Identical	Complete	Genetic confirmation of clinical diagnosis
**02795**	No	Blue cone monochromacy	1	2	Identical	Complete	Genetic confirmation of clinical diagnosis
**02802**	No	Bornholm eye disease	1	2	Identical	Complete	Genetic confirmation of clinical diagnosis
**03135**	No	Blue cone monochromacy	1	3	Identical	Complete	No genetic confirmation of clinical diagnosis
**03161**	Yes	Bornholm eye disease	1	1	Identical	Complete	Genetic confirmation of clinical diagnosis
**03167**	No	Blue cone monochromacy	1	3	Identical	Complete	No genetic confirmation of clinical diagnosis
**03197**	Yes	Bornholm eye disease	1	2	Identical	Complete	Genetic confirmation of clinical diagnosis

**15524**	Yes	Bornholm eye disease	1	3	Different	Complete	Genetic confirmation of clinical diagnosis
**03473**	No	Protanopia	1	1	Identical	Complete	Genetic confirmation of clinical diagnosis
**03517**	No	Myopia	1	2	Identical	Complete	Unclear whether genetic results confirm clinical diagnosis, due to the presence of (a) variant(s) of uncertain significance
**03582**	No	Bornholm eye disease	1	2	Identical	Complete	Genetic confirmation of clinical diagnosis
**03819**	No	Cone dystrophy	1	1	Identical	Complete	Genetic confirmation of clinical diagnosis
**03842**	No	Blue cone monochromacy	1	3	Identical	Complete	Genetic confirmation of clinical diagnosis
**04311**	No	Blue cone monochromacy	1	1	Identical	Complete	Genetic confirmation of clinical diagnosis
**04332**	No	Bornholm eye disease	1	2	Identical	Complete	Unclear whether genetic results confirm clinical diagnosis, due to the presence of (a) variant(s) of uncertain significance
**04415**	No	Myopia	1	2	Identical	Complete	Unclear whether genetic results confirm clinical diagnosis, due to the presence of (a) variant(s) of uncertain significance
**04460**	No	Bornholm eye disease	1	1	Identical	Complete	Genetic confirmation of clinical diagnosis
**04466**	Yes	Blue cone monochromacy	0	0	Identical	Complete	Genetic confirmation of clinical diagnosis
**04594**	No	Bornholm eye disease	1	3	Identical	Complete	No genetic confirmation of clinical diagnosis
**04606**	No	Blue cone monochromacy	1	2	Identical	Complete	Genetic confirmation of clinical diagnosis
**04683**	No	Deuteranopia	1	1	Identical	Complete	Genetic confirmation of clinical diagnosis
**04714**	No	Blue cone monochromacy	1	4	Different	Incomplete	Unclear whether genetic results confirm clinical diagnosis, as it was not possible to determine complete composition of *OPN1LW*/*OPN1MW* gene cluster
**04813**	No	Bornholm eye disease	1	4	Identical	Complete	No genetic confirmation of clinical diagnosis
**04819**	No	Blue cone monochromacy	1	4	Identical	Complete	No genetic confirmation of clinical diagnosis
**04847**	No	Blue cone monochromacy	1	2	Identical	Complete	Genetic confirmation of clinical diagnosis
**04934**	No	Blue cone monochromacy	1	1	Identical	Complete	Genetic confirmation of clinical diagnosis
**05330**	No	Bornholm eye disease	1	2	Identical	Complete	No genetic confirmation of clinical diagnosis
**05622**	No	Bornholm eye disease	1	5	Identical	Complete	No genetic confirmation of clinical diagnosis
**05773**	No	Bornholm eye disease	1	2	Identical	Complete	No genetic confirmation of clinical diagnosis
**06120**	No	Blue cone monochromacy	1	5	Identical	Complete	Genetic confirmation of clinical diagnosis
**06164**	Yes	Bornholm eye disease	1	3	Identical	Complete	Genetic confirmation of clinical diagnosis
**06204**	No	Protanopia	1	1	Identical	Complete	Genetic confirmation of clinical diagnosis
**06305**	No	Bornholm eye disease	1	3	Identical	Complete	No genetic confirmation of clinical diagnosis
**06413**	Yes	Blue cone monochromacy	1	1	Identical	Complete	Genetic confirmation of clinical diagnosis
**06537**	Yes	Bornholm eye disease	1	2	Identical	Complete	Genetic confirmation of clinical diagnosis
**06648**	No	Bornholm eye disease	1	3	Identical	Complete	No genetic confirmation of

							clinical diagnosis
**06650**	No	Bornholm eye disease	1	3	Identical	Complete	No genetic confirmation of clinical diagnosis
**07403**	Yes	Protanopia	1	2	Identical	Complete	Genetic confirmation of clinical diagnosis
**18156**	No	Blue cone monochromacy	1	3	Different	Incomplete	No genetic confirmation of clinical diagnosis
**07788**	No	Myopia	1	3	Identical	Complete	Unclear whether genetic results confirm clinical diagnosis, due to the presence of (a) variant(s) of uncertain significance
**08043**	Yes	Blue cone monochromacy	1	2	Identical	Complete	Genetic confirmation of clinical diagnosis
**08246**	No	Blue cone monochromacy	1	1	Identical	Complete	Genetic confirmation of clinical diagnosis
**08449**	No	Myopia	1	3	Identical	Complete	No genetic confirmation of clinical diagnosis
**08625**	No	Myopia	1	2	Identical	Complete	Genetic confirmation of clinical diagnosis
**08627**	Yes	Bornholm eye disease	1	1	Identical	Complete	Genetic confirmation of clinical diagnosis
**09028**	No	Blue cone monochromacy	1	3	Identical	Complete	No genetic confirmation of clinical diagnosis
**09902**	No	Myopia	1	3	Identical	Complete	Unclear whether genetic results confirm clinical diagnosis, due to the presence of (a) variant(s) of uncertain significance
**09955**	Yes	Bornholm eye disease	1	2	Identical	Complete	Genetic confirmation of clinical diagnosis
**10102**	No	Myopia	1	2	Identical	Complete	No genetic confirmation of clinical diagnosis
**10154**	Yes	Blue cone monochromacy	1	1	Identical	Complete	Genetic confirmation of clinical diagnosis
**10256**	No	Bornholm eye disease	1	2	Identical	Complete	Genetic confirmation of clinical diagnosis
**10272**	No	Bornholm eye disease	1	2	Identical	Complete	Genetic confirmation of clinical diagnosis
**03277**	No	Bornholm eye disease	1	3	Different	Complete	Genetic confirmation of clinical diagnosis
**11264**	No	Bornholm eye disease	1	2	Identical	Complete	Genetic confirmation of clinical diagnosis
**11290**	Yes	Bornholm eye disease	1	2	Identical	Complete	Genetic confirmation of clinical diagnosis
**11817**	No	Bornholm eye disease	1	4	Identical	Complete	Genetic confirmation of clinical diagnosis
**11990**	No	Cone dystrophy	1	3	Identical	Complete	No genetic confirmation of clinical diagnosis
**12011**	No	Myopia	1	2	Identical	Complete	Unclear whether genetic results confirm clinical diagnosis, due to the presence of (a) variant(s) of uncertain significance
**12149**	No	Bornholm eye disease	1	3	Identical	Complete	Unclear whether genetic results confirm clinical diagnosis, due to the presence of (a) variant(s) of uncertain significance
**11008**	No	Bornholm eye disease	1	3	Different	Complete	Genetic confirmation of clinical diagnosis
**12430**	No	Blue cone monochromacy	1	3	Identical	Complete	Genetic confirmation of clinical diagnosis
**12516**	No	Cone dystrophy	1	4	Identical	Complete	No genetic confirmation of clinical diagnosis
**12646**	No	Bornholm eye disease	1	2	Identical	Complete	Genetic confirmation of clinical diagnosis
**12850**	No	Bornholm eye disease	1	2	Identical	Complete	Genetic confirmation of clinical diagnosis
**12911**	No	Bornholm eye disease	1	4	Different	Incomplete	Genetic confirmation of clinical diagnosis

**13108**	Yes	Bornholm eye disease	1	2	Identical	Complete	Genetic confirmation of clinical diagnosis
**16493**	No	Bornholm eye disease	1	3	Different	Complete	Genetic confirmation of clinical diagnosis
**13166**	No	Blue cone monochromacy	1	1	Identical	Complete	Genetic confirmation of clinical diagnosis
**13609**	Yes	Bornholm eye disease	1	3	Identical	Complete	Genetic confirmation of clinical diagnosis
**13612**	No	Bornholm eye disease	1	4	Identical	Complete	Genetic confirmation of clinical diagnosis
**13677**	No	Bornholm eye disease	1	1	Identical	Complete	Genetic confirmation of clinical diagnosis
**13702**	No	Bornholm eye disease	1	2	Identical	Complete	Genetic confirmation of clinical diagnosis
**13808**	Yes	Bornholm eye disease	1	3	Identical	Complete	Genetic confirmation of clinical diagnosis
**14217**	No	Blue cone monochromacy	1	1	Identical	Complete	Genetic confirmation of clinical diagnosis
**14288**	No	Blue cone monochromacy	1	2	Identical	Complete	No genetic confirmation of clinical diagnosis
**14345**	Yes	Bornholm eye disease	1	4	Identical	Complete	Genetic confirmation of clinical diagnosis
**14425**	No	Blue cone monochromacy	1	2	Identical	Complete	Genetic confirmation of clinical diagnosis
**00750**	No	Bornholm eye disease	1	3	Different	Complete	Genetic confirmation of clinical diagnosis
**14728**	No	Blue cone monochromacy	1	2	Identical	Complete	Genetic confirmation of clinical diagnosis
**15024**	Yes	Blue cone monochromacy	0	3	Identical	Complete	Genetic confirmation of clinical diagnosis
**15088**	No	Bornholm eye disease	1	3	Identical	Complete	No genetic confirmation of clinical diagnosis
**15155**	No	Blue cone monochromacy	1	1	Identical	Complete	Genetic confirmation of clinical diagnosis
**15176**	No	Bornholm eye disease	1	2	Identical	Complete	Genetic confirmation of clinical diagnosis
**15189**	No	Bornholm eye disease	1	3	Identical	Complete	No genetic confirmation of clinical diagnosis
**15375**	No	Blue cone monochromacy	1	3	Identical	Complete	No genetic confirmation of clinical diagnosis
**15384**	No	Bornholm eye disease	1	3	Identical	Complete	Unclear whether genetic results confirm clinical diagnosis, due to the presence of (a) variant(s) of uncertain significance
**15406**	Yes	Blue cone monochromacy	1	1	Identical	Complete	Genetic confirmation of clinical diagnosis
**12334**	No	Bornholm eye disease	1	3	Different	Complete	Genetic confirmation of clinical diagnosis
**15585**	Yes	Blue cone monochromacy	1	1	Identical	Complete	Genetic confirmation of clinical diagnosis
**15705**	No	Blue cone monochromacy	1	1	Identical	Complete	Genetic confirmation of clinical diagnosis
**15706**	No	Blue cone monochromacy	1	2	Identical	Complete	Genetic confirmation of clinical diagnosis
**15792**	No	Cone dystrophy	1	2	Identical	Complete	Genetic confirmation of clinical diagnosis
**15983**	Yes	Bornholm eye disease	1	3	Identical	Complete	No genetic confirmation of clinical diagnosis
**16131**	No	Bornholm eye disease	1	3	Identical	Complete	No genetic confirmation of clinical diagnosis
**16251**	No	Bornholm eye disease	1	3	Identical	Complete	Unclear whether genetic results confirm clinical diagnosis, due to the presence of (a) variant(s) of uncertain significance
**01949**	No	Bornholm eye disease	1	3	Different	Incomplete	No genetic confirmation of clinical diagnosis
**16633**	No	Blue cone monochromacy	1	2	Identical	Complete	Genetic confirmation of clinical diagnosis
**16975**	No	Bornholm eye disease	1	3	Identical	Complete	No genetic confirmation of

							clinical diagnosis
**16986**	No	Bornholm eye disease	1	1	Identical	Complete	Genetic confirmation of clinical diagnosis
**17035**	No	Bornholm eye disease	1	4	Identical	Complete	No genetic confirmation of clinical diagnosis
**17179**	No	Blue cone monochromacy	1	3	Identical	Complete	No genetic confirmation of clinical diagnosis
**17203**	No	Blue cone monochromacy	1	3	Identical	Complete	Genetic confirmation of clinical diagnosis
**17877**	No	Blue cone monochromacy	1	2	Identical	Complete	No genetic confirmation of clinical diagnosis
**18048**	No	Bornholm eye disease	1	3	Identical	Complete	No genetic confirmation of clinical diagnosis
**07743**	No	Cone dystrophy	1	3	Different	Complete	No genetic confirmation of clinical diagnosis
**18180**	Yes	Blue cone monochromacy	1	3	Identical	Complete	Genetic confirmation of clinical diagnosis
**18340**	Yes	Blue cone monochromacy	1	2	Identical	Complete	Genetic confirmation of clinical diagnosis
**18483**	No	Blue cone monochromacy	1	1	Identical	Complete	Genetic confirmation of clinical diagnosis
**18507**	No	Bornholm eye disease	1	2	Identical	Complete	No genetic confirmation of clinical diagnosis
**18715**	No	Blue cone monochromacy	1	2	Identical	Complete	Genetic confirmation of clinical diagnosis
**19224**	No	Bornholm eye disease	1	2	Identical	Complete	Genetic confirmation of clinical diagnosis
**19411**	No	Cone dystrophy	1	3	Identical	Complete	No genetic confirmation of clinical diagnosis
**19423**	No	Blue cone monochromacy	1	3	Identical	Complete	No genetic confirmation of clinical diagnosis
**19669**	No	Deuteranopia	1	1	Identical	Complete	Genetic confirmation of clinical diagnosis
**20083**	Yes	Bornholm eye disease	1	3	Identical	Complete	No genetic confirmation of clinical diagnosis
**20249**	No	Blue cone monochromacy	1	3	Identical	Complete	No genetic confirmation of clinical diagnosis
**02213**	No	Cone dystrophy	1	3	Different	Complete	Genetic confirmation of clinical diagnosis
**20480**	Yes	Bornholm eye disease	1	3	Identical	Complete	Genetic confirmation of clinical diagnosis
**20647**	No	Blue cone monochromacy	1	1	Identical	Complete	Genetic confirmation of clinical diagnosis
**21103**	No	Blue cone monochromacy	1	1	Identical	Complete	Genetic confirmation of clinical diagnosis
**21190**	No	Bornholm eye disease	1	2	Identical	Complete	Genetic confirmation of clinical diagnosis
**21260**	No	Blue cone monochromacy	1	1	Identical	Complete	Genetic confirmation of clinical diagnosis
**21386**	No	Blue cone monochromacy	1	2	Identical	Complete	No genetic confirmation of clinical diagnosis
**21479**	No	Bornholm eye disease	1	3	Identical	Complete	Genetic confirmation of clinical diagnosis
**21614**	No	Bornholm eye disease	1	1	Identical	Complete	Genetic confirmation of clinical diagnosis
**22414**	No	Myopia	1	2	Identical	Complete	No genetic confirmation of clinical diagnosis
**22692**	No	Bornholm eye disease	1	3	Identical	Complete	No genetic confirmation of clinical diagnosis
**22812**	No	Myopia	1	4	Identical	Complete	Unclear whether genetic results confirm clinical diagnosis, due to the presence of (a) variant(s) of uncertain significance
**23212**	No	Myopia	1	2	Identical	Complete	No genetic confirmation of clinical diagnosis
**23344**	No	Bornholm eye disease	1	2	Identical	Complete	Genetic confirmation of clinical diagnosis

**23395**	No	Bornholm eye disease	1	2	Identical	Complete	Unclear whether genetic results confirm clinical diagnosis, due to the presence of (a) variant(s) of uncertain significance
**30052**	No	Blue cone monochromacy	1	3	Identical	Complete	No genetic confirmation of clinical diagnosis
**31808**	Yes	Bornholm eye disease	1	2	Identical	Complete	Genetic confirmation of clinical diagnosis
**35591**	Yes	Blue cone monochromacy	1	1	Identical	Complete	Genetic confirmation of clinical diagnosis

Supplementary Table 2. Quality values of long-read genome sequencing

	**>= Q20 reads**	**Yield**	**Mean Length**	**Median QV**	**coverage genome-wide**	**coverage chrX**
**USN 15600**	6680341	98.22 Gb	14703	Q33	30.41x	16.88x
**USN 15524**	6769782	103.36 Gb	15267	Q32	31.97x	17.78x

Supplementary Figure 1

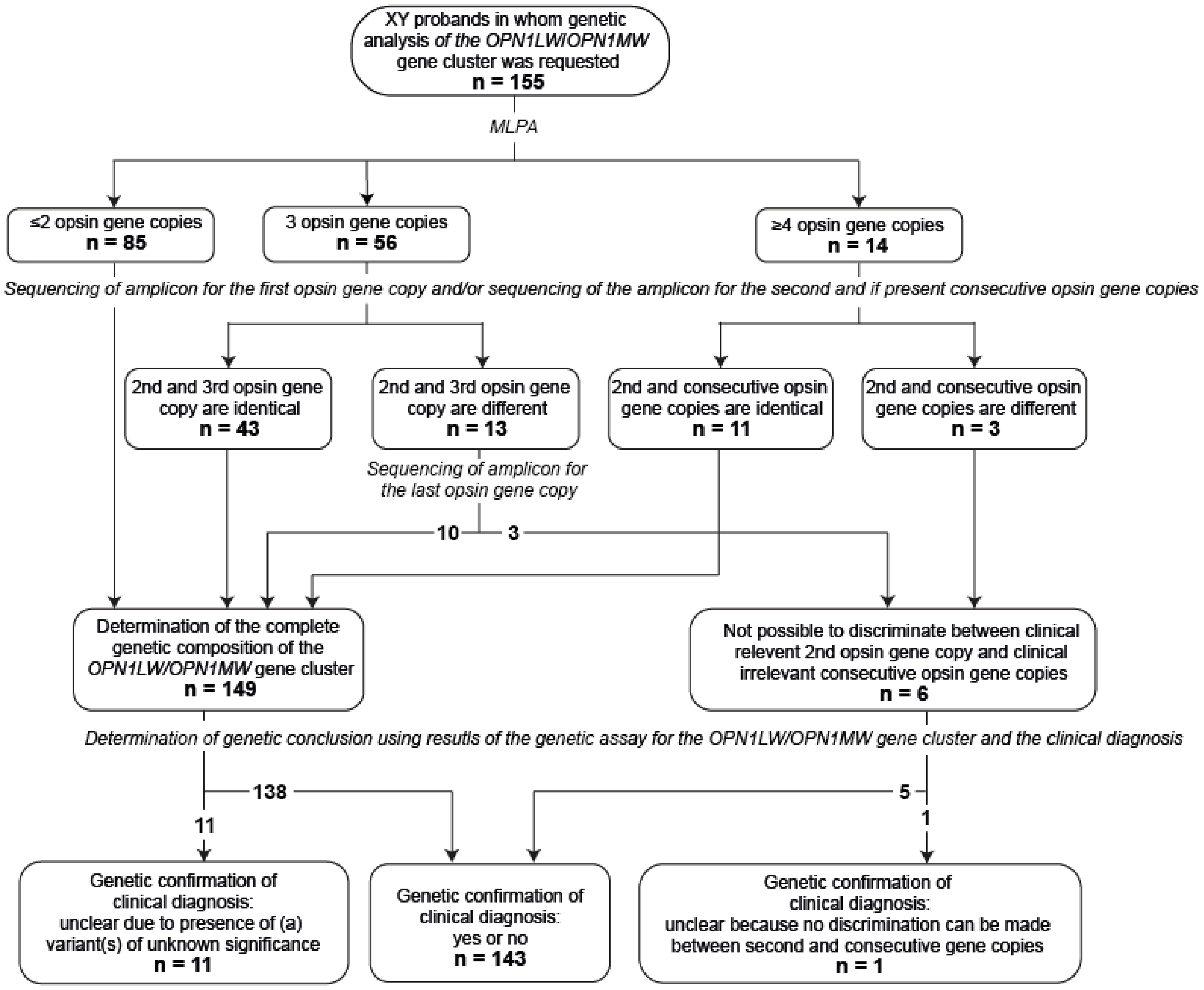

Supplementary Figure 1. Flow diagram of results of the genetic analysis of the *OPN1LW/OPN1MW* gene cluster in 155 XY probands. With MLPA analysis the number of opsin gene copies is determined. In 85 probands two or less opsin gene copies, in 56 probands three opsin gene copies and in 14 probands four or more opsin gene copies were detected.

In all 85 probands with two or less opsin gene copies, the complete genetic composition of the opsin gene cluster can be determined via sequencing of the amplicon for the first opsin gene copy and/or the amplicon for the second and if present consecutive opsin gene copies.

In 43 of the 56 probands with three opsin gene copies, the amplicon for the second and if present consecutive opsin gene copies determined that the second and third opsin gene copy were identical and therefore also in these individuals the assay determined the complete composition of the *OPN1LW/OPN1MW* gene cluster. In 13 of the 56 probands with three opsin gene copies, the

amplicon for the second and if present consecutive opsin gene copies determined that the second and third opsin gene copy were not identical. Sequencing of the amplicon for the last opsin gene in the cluster was performed in all 13 probands. Taken the results of the sequencing of the last opsin gene copy together with the distribution of the SDIns in 3 gene copy alleles as shown in Figure 2, we assume that 8 probands had a composition as depicted in possibility a in Figure 2 (this assumed composition was confirmed in 2 probands with long-read genome sequencing), 2 had a composition as depicted in possibility b in Figure 2, 1 had a composition as depicted in possibility c in Figure 2 and 2 had a composition as depicted in possibility d in Figure 2. In the latter three probands we were thus unable to determine the complete composition of the *OPN1LW*/*OPN1MW* gene cluster, while in the first ten probands we assume that we can correctly determine the complete composition of the opsin gene cluster.

In 3 of the 14 probands with four or more opsin gene copies the second and consecutive opsin gene copies where not identical, therefore the assay could not determine the complete composition of the *OPN1LW*/*OPN1MW* gene cluster, while in 11 the gene copies were identical and the assay could determine the complete composition.

The results of the genetic assay in combination with the clinical diagnosis are combined to make a genetic conclusion. It is not always necessary to determine the complete composition of the *OPN1LW*/*OPN1MW* gene cluster to come to a genetic diagnosis, as shown for 5 out of the 6 in whom it was possible to make a genetic conclusion, while it was not possible to determine the complete composition of the *OPN1LW/OPN1MW* gene cluster. Only in 1 of 155 probands (this proband had 4 opsin gene copies in total) the genetic conclusion was unclear because the composition of the *OPN1LW*/*OPN1MW* gene cluster could not be determined. This uncertainness was reported back to the requesting physician, and therefore also in this case no incorrect genetic conclusion was reported.

Supplementary Figure 2

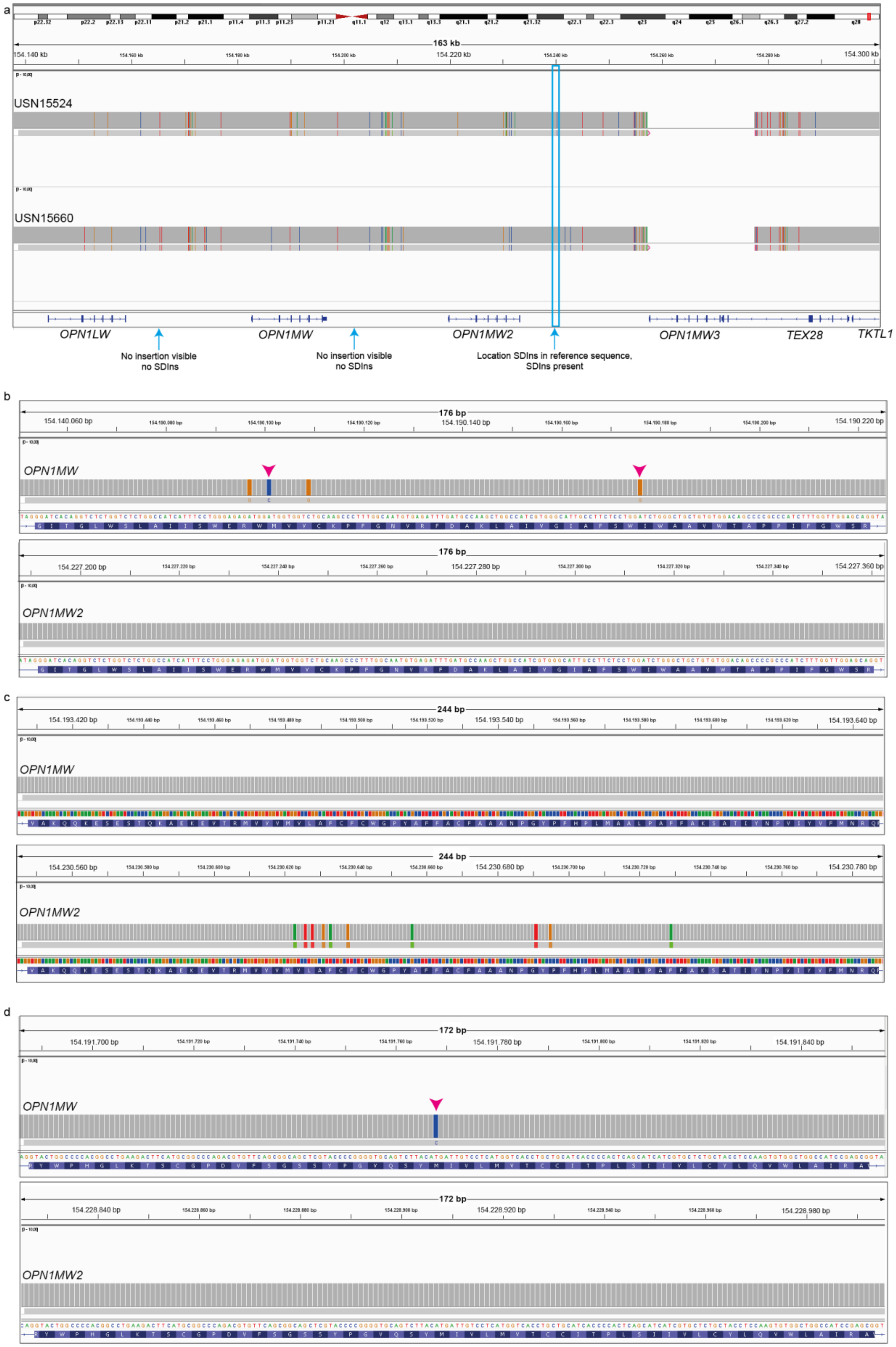

Supplementary Figure 2: Print screens of IGV of the *OPN1LW/OPN1MW* gene cluster of *de novo* assembly of long-read genome sequencing data of samples USN15524 and USN15600. The sequencing data is aligned to genome build GRCh38. Because of the *de novo* assembly only the single detected haplotype is shown. In both samples the long-read genome sequencing confirms the results determined with the amplicon for the last opsin gene copy of the cluster. **a**) Overview of the complete *OPN1LW/OPN1MW* gene cluster. Both samples consist of three opsin gene copies and a gap is seen in the sequence at the position of the fourth opsin gene copy (*OPN1MW3*) that is present in reference sequence of GRCh38. The sequencing data show that the second and third opsin gene copy are genetically different, which was also determined by the genetic assay specific for the *OPN1LW/OPN1MW* gene cluster. In the GRCh38 reference genome the SDIns is solely located after the third opsin gene copy, named *OPN1MW2*. In both USN15524 and USN15660 the SDIns was present after the third opsin gene copy, represented by the sequence within the blue rectangle, while no 697 bp insertions was detected after the *OPN1LW* and/or *OPN1MW* gene in both samples. Thus the SDIns was solely present after the last opsin gene copy in both samples. **b**) Overview of exons 3 of the second and third gene copy in the gene cluster (*OPN1MW*, top and *OPN1MW2*, bottom) for sample USN15524. The LVAVA pathogenic combination of variants (the two differences of the LVAVA combination compared to the reference are indicated with pink arrow-heads) is present in exon 3 of the second (clinical relevant) opsin gene copy. **c**) Overview of exons 5 of the second and third gene copy in the gene cluster (*OPN1MW*, top and *OPN1MW2*, bottom) for sample USN15524. In this sample, the third (clinically irrelevant) opsin gene is a hybrid *OPN1MW-OPN1LW* gene, where *OPN1MW* exon 5 has been exchanged with exon 5 of *OPN1LW*. **d**) Overview of exons 4 of the second and third gene copy in the gene cluster (*OPN1MW*, top and *OPN1MW2*, bottom) for sample USN15600. The c.659T>C p.(Met220Thr) variant of unknown significance (indicated with pink arrow-head) is present in exon 4 of the second (clinical relevant) opsin gene copy.

Supplementary Figure 3

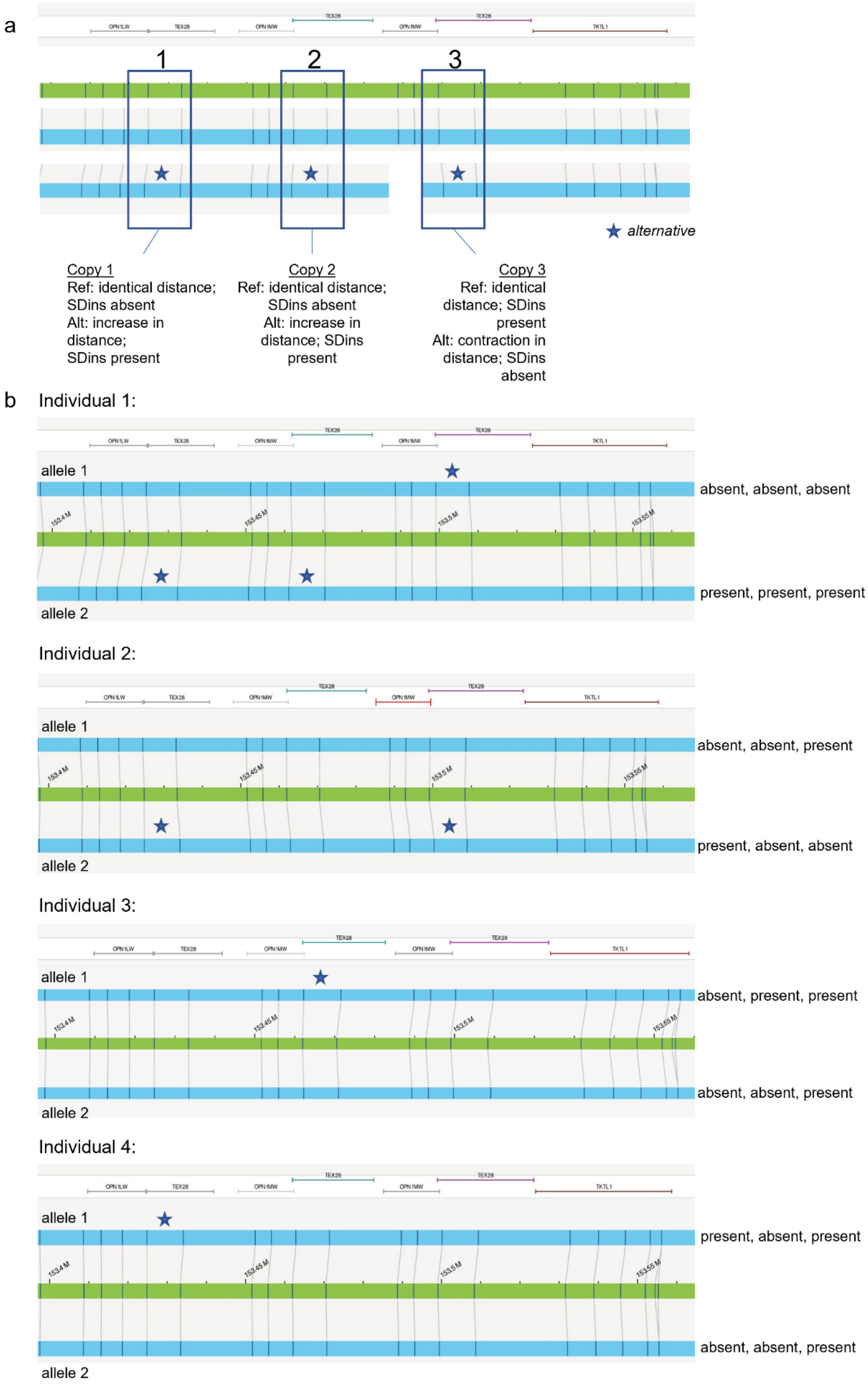

Supplementary Figure 3. Optical genome mapping data representing the *OPN1LW/MW* gene cluster containing one *OPN1LW* gene copy and two *OPN1MW* gene copies, as given in reference GRCh37/hg19. a) Illustration of the variant calling for the three different opsine gene copies using optical genome mapping. In case the SDIns is present downstream of *OPN1LW* (copy 1) or *OPN1MW* (copy 2), the Bionano Access software will identify an increase of the distance between the labels and result in a corresponding call of an insertion. No call at the respective position means that the SDIns is not present at that location. Concerning *OPN1MW2* (copy 3) the situation is different, as here the SDIns is already included in the reference sequence. Accordingly, no call means that the SDIns is present, whereas the absence of the SDIns results in the call of a deletion. b) Four different examples are shown to illustrate six different haploypes that harbor the 697bp SDIns at the three different genomic locations (downstream of *OPN1LW*, downstream of *OPN1MW*, downstream of *OPN1MW2*, or a combination of those). In total, 8 different combinations of the SDIns are theoretically expected. Six of those are shown here. The additional 2, absent-present-absent and present-present-absent were not detected in a cohort of 200 alleles harboring three opsine gene copies. Ref: reference; Alt: alternative.

REFERENCES

1. Haer-Wigman L, den Ouden A, van Genderen MM, Kroes HY, Verheij J, Smailhodzic D, et al. Diagnostic analysis of the highly complex OPN1LW/OPN1MW gene cluster using long-read sequencing and MLPA. NPJ Genom Med. 2022;7(1):65.
2. Kucuk E, van der Sanden B, O'Gorman L, Kwint M, Derks R, Wenger AM, et al. Comprehensive de novo mutation discovery with HiFi long-read sequencing. Genome Med. 2023;15(1):34.
